# Anti-Human Immunodeficiency Virus-1 Property of Thai Herbal Extract Kerra™

**DOI:** 10.3390/ph17070917

**Published:** 2024-07-09

**Authors:** Siriwan Saehlee, Supaphorn Seetaha, Wiwat Klankaew, Pussadee Srathong, Kiattawee Choowongkomon, Khuanjarat Choengpanya

**Affiliations:** 1 Department of Biochemistry, Faculty of Science, Kasetsart University, Bangkok 10900, Thailand; siriwan.sah08@gmail.com (S.S.); supaporn.se@ku.th (S.S.); 2 Interdisciplinary of Genetic Engineering and Bioinformatics, Graduate School, Kasetsart University, Bangkok 10900, Thailand; wiwat.kl@ku.th; 3 Faculty of Nursing, Praboromarajchanok Institute, Nonthaburi 11000, Thailand; pussadee10@yahoo.com; 4 Program in Basic Science, Maejo University–Phrae Campus, Phrae 54140, Thailand

**Keywords:** HIV-1, reverse transcriptase, Thai herbal extract, molecular docking

## Abstract

Kerra™, a Thai traditional herbal medicine derived from the “Tak-Ka-Si-La Scripture” and composed of nine medicinal plants, has demonstrated potential antiviral properties against HIV. This study investigated the inhibitory effects of Kerra™ on HIV-1 reverse transcriptase (RT) and its ability to prevent pseudo-HIV viral infection in HEK293 cells. The results showed that Kerra™ extract achieved a 95.73 ± 4.24% relative inhibition of HIV-1 RT, with an IC_50_ value of 42.66 ± 8.74 µg/mL. Docking studies revealed that key phytochemicals in Kerra™, such as oleamide, formononetin, and biochanin A, interact with several residues in the RT non-nucleoside binding pocket, contributing to their inhibitory effects. Furthermore, Kerra™ was able to reduce pseudo-HIV infection in HEK293 cells at a concentration of 10 µg/mL, suggesting its potential as a supplementary treatment for HIV.

## 1. Introduction

RNA viruses are considered a major cause of infectious diseases in humans and animals. One of the major RNA viruses in humans is human immunodeficiency virus (HIV), which is a single-stranded RNA virus causing acquired immunodeficiency syndrome (AIDS). In 2022, approximately 630,000 people died from HIV-related causes, and about 39.0 million people are living with HIV [1]. HIV has high mutation and recombination rates, and the interaction between HIV and the host immune response have made the development of vaccines challenging [2,3,4]. So far, many clinical trials of HIV vaccines have been conducted. Some trials failed to raise a protective response, and some trials were discontinued due to safety concerns and inefficiency in preventing HIV transmission. A few clinical trials finished the tests but showed a low protective rate (31.2%) [3,5]. These failures of the HIV vaccine tests have made the antiretroviral therapy (ART) the only effective therapy for HIV-infected individuals.

ART is a combination of antiviral compounds targeting different HIV enzymes and proteins, such as reverse transcriptase, protease, integrase, and proteins, involving viral binding and entry [4,6]. ART has been successfully used to suppress the viral load, increase the CD4+ level, and decrease the mortality rate and incidence of opportunistic illness [7]. An example of FDA-approved drugs used to treat HIV-positive patients is the combination of emtricitabine, efavirenz, and tenofovir disoproxil fumarate, which inhibit HIV-1 reverse transcriptase (HIV-1 RT) [6]. HIV-1 RT is an important enzyme, responsible for the synthesis of proviral double-stranded DNA from its RNA, leading to the replication cycle of viral DNA in infected cells [4]. Emtricitabine and tenofovir disoproxil fumarate are nucleotide/nucleoside reverse transcriptase inhibitors (NRTIs), and their structures resemble natural nucleotides, except that they lack a 3′-hydroxyl group [8,9]. The drugs compete with the natural nucleotides to bind into the polymerase active site of HIV-1 RT. Once the NRTIs are incorporated into the growing viral DNA strand, the termination of viral DNA synthesis occurs. Efavirenz is one of the non-nucleoside reverse transcriptase inhibitors (NNRTIs). The structures of NNRTIs are varied. NNRTIs non-competitively bind into the non-nucleoside binding pocket (NNBP), which is approximately 10 Å away from the polymerase active site. The binding of NNRTIs causes a restriction of the conformational change in HIV-1 RT, resulting in allosteric inhibition of HIV-1 RT [8]. Although ART has been successfully used to treat HIV-infected individuals, the long-term use of ART can cause side effects and the emergence of resistant HIV-1 variants [6,9,10,11]. SeyedAlinaghi et al. (2023) performed an umbrella review, focusing on virologic failure, and revealed that ART with NNRTIs showed more frequent effects of drug resistance than other types of ART [11]. Therefore, the screening for novel NNRTIs is necessary to fight against the resistant variants.

Medicinal plants are rich sources of phytochemicals with therapeutic properties, including antioxidant, antimicrobial, anti-inflammatory, anticancer, and antiviral properties [12,13,14,15,16]. Various plant species have been used to study the efficacy of plant extracts in the treatment of viral infectious diseases [14,15,16,17,18,19,20]. Abdel-Malek et al. (1996) screened 60 plant species for anti-HIV activity and reported that 18 plant species, e.g., *Achyrocline alata*, *Piper elongatum*, and *Xanthium spinosum*, exhibited protective activity for MT-2 T-lymphoblastoid cells from the cytopathic effect of HIV [17]. Seventeen aqueous and methanol extracts from nine South African medicinal plants were screened for anti-HIV-1 RT, and the results showed that almost all the extracts, except the aqueous extracts of *Mucuna coriacea*, *Peltophorum africanum*, and *Vernonia stipulacea*, exhibited various anti-HIV-1 RT activities, with IC_50_ values ranging between 3.5 and >2000 µg/mL [18]. Methanolic extracts from the roots of four species of *Thymus*, namely *T. vulgaris* L., *T. kotschyanus* Boiss. & Hohen., *T. carmanicus* Jalas, and *T. daenensis* Celak, showed anti-HIV-1 replication activity [19]. Dichloromethane extract from the stem bark of *Erythrina senegalensis* exhibited HIV-1 protease inhibition activity [20]. Some phytochemicals with anti-HIV-1 activity have been isolated and identified, and it was found that these phytochemicals were terpenes and terpenoid lignan and coumarin [15,16]. For example, gallotannin isolated from methanolic extract of *P. africanum* inhibited HIV-1 RT with an IC_50_ value of 6.0 µM [18]. 8,8a-epoxymorellic acid, which was isolated from ethyl acetate extract of *Garcinia hanburyi*, inhibited HIV-1 RT with an IC_50_ value of 101.8 µg/mL [20]. Eight prenylisoflavonoids, namely 8-prenylluteone, auriculatin, erysenegalensein O, erysenegalensein D, erysenegalensein N, derrone, alpinumisoflavone, and 6,8-diprenylgenistein, were responsible for anti-HIV-1 protease activity in dichloromethane extract of *E. senegalensis* [21]. Plant extracts not only inhibit the key enzymes in the viral life cycle, they also have immunological effects on HIV-infected patients. It has been shown that a combination of ART and aqueous extract from the leaves of *Vernonia amygdalina* could increase the CD4 cell count [22].

Kerra™ is a Thai traditional herbal medicine developed from the ancient Thai scripture “Tak-Ka-Si-La Scripture”. It is already registered by the Thai Food and Drug Administration. Kerra™ consists of nine medicinal plants, which are *Pterocarpus santalinus* L.f., *Mansonia gagei* J.R. Drumm. ex Prai., *Schumannianthus dichotomus* (Roxb.) Gagnep., *Momordica cochinchinensis* (Lour.) Spreng, *Citrus aurantifolia* (Christm.) Swingle, *Combretum quadrangutare* Kurz, *Tiliacora triandra* (Colebr.) Diels, *Tinospora crispa* (L.) Miers ex Hook.f. and Thoms, and *Dregea volubilis* (l.f.) Hook.f. [12]. Some of these medicinal plants have been shown to possess antiviral properties [23,24]. Pterostilbene, a phytochemical that is present in *P. santalinus* L.f., was found to entirely prevent HIV-1 infection in resting CD4 T cells at the reverse transcription step [25]. The methanolic crude extract of *M. cochinchinensis* showed 22.4 ± 7.4% inhibition against HIV-1 protease at a concentration of 100 µg/mL [26]. The phytochemical profile of Kerra™ was previously identified by the LC-MS/MS technique, and 414 phytochemicals were identified. 2-Methoxy-9H-xanthen-9-one, isorhapontigenin, betaine, *trans*-anethole, eicosatetraynoic acid, NP-020078, NP-003294, and N1-(3-chlorophenyl)-2-[2-(trifluoromethyl)-4-quinolyl]hydrazine-1-carboxamide were the top nine phytochemicals found in the extract, with the peak areas ranging between 2.3 × 10^9^ and 7.43 × 10^9^ [12]. Our previous study showed that Kerra™ had inhibitory effects against SARS-CoV-2 and human papillomavirus (HPV) viruses [12,13] In the current study, we explored the effects of Kerra™ against another RNA virus, HIV-1. The anti-reverse transcriptase and inhibition of pseudo-HIV infection properties of Kerra™ against wild-type HIV was investigated.

## 2. Results and Discussion

### 2.1. Inhibition Study

**Figure 1:**
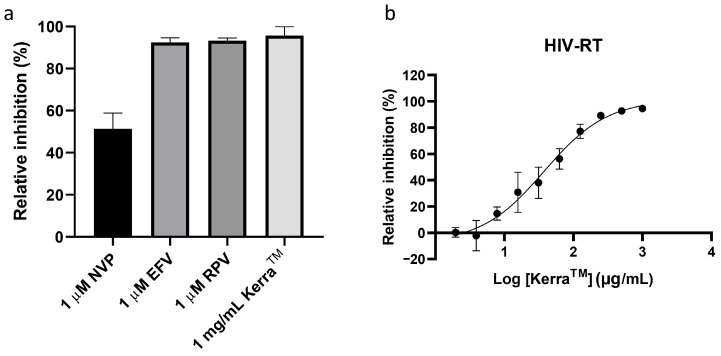
Inhibition study. (**a**) Percentage relative inhibition of three drugs, which were nevirapine (NVP), efavirenz (EFV), and rilpivirine (RPV), and Kerra™ extract against HIV-1 RT; (**b**) non−linear regression dose−response curve of Kerra™ extract against HIV-1 RT.

The percentage of relative inhibitions of the three FDA-approved drugs, nevirapine (NVP), efavirenz (EFV), and rilpivirine (RPV), and Kerra™ extract against HIV-1 RT are shown in Figure 1a. The Kerra™ extract could inhibit HIV-1 RT with a relative inhibition percentage of 95.73 ± 4.24. According to the one-way ANOVA analysis (*p* < 0.05), the results showed that at least one pair of samples was significantly different. After applying the Bonferroni correction (α = 0.0083) in a post hoc test, it was found that the relative inhibition percentage of Kerra™ was significantly different to NVP, but not to EFV and RPV. Kerra™ demonstrated superior inhibition of HIV-1 RT compared to NVP.

The determination of the IC_50_ value of Kerra™ was performed. The IC_50_ value of Kerra™ extract against HIV-1 RT was 38.00 ± 4.62 µg/mL (Figure 1b and Table 1). The IC_50_ value of Kerra™ was higher than that of EFV and other plant extracts, such as *Bridelia micrantha* (IC_50_ = 18.5 µg/mL) and *Combretum molle* (IC_50_ = 9.5 µg/mL). It had a similar IC_50_ value to *Peltophorum africanum* (IC_50_ = 38.3 µg/mL) [18]. However, Kerra™ had greater inhibition activity than extracts of *G. hanburyi* (IC_50_ = 101.8 µg/mL), *Ricinus communis* (IC_50_ = 182.0 µg/mL), *Sutherlandia frutescens* (IC_50_ = 425.0 µg/mL), *Harungana madagascariensis* (IC_50_ = 900 µg/mL), *Sapium ellipticum* (IC_50_ = 1.05 mg/mL), and *Pseudospondias microcarpa* (IC_50_ = 1.82 mg/mL), [18,20,27]. The variations in IC_50_ values might be attributed to the different phytochemical constituents [18]. The results of the inhibition experiments revealed that Kerra™ could inhibit reverse transcriptase, a crucial enzyme of HIV-1. The phytochemicals responsible for the HIV-1 RT inhibition effect were subsequently investigated using an in silico approach.

### 2.2. Molecular Docking

To investigate the phytochemicals of Kerra™, which might be responsible for the anti-HIV-1 RT activity, molecular docking was used. The HIV-1 RT enzyme is known to be a heterodimer consisting of two protein subunits, p51 and p66. The p66 subunit is flexible and can be repositioned upon binding to DNA, nucleotides, or anti-RT drugs [29]. Three anti-HIV-1 RT drugs, namely NVP, EFV, and RPV, were docked into the NNBP of HIV-1 RT. The docking results are shown in Table 2. In the case of Kerra™ extract, its phytochemical profile was analyzed using the LC-MS/MS technique, identifying 414 phytochemical species [12]. The structures of the 31 most abundant phytochemicals were searched in the PubChem and AnalytiCon Discovery databases, and 21 structures were obtained. The docking results for these 21 structures are presented in Table 2.

The docking of the three FDA-approved drugs, NVP, EFV, and RPV, into the NNBP of HIV-1 RT revealed that RPV had the highest GOLD docking scores, followed by EFV and NVP. When docking Kerra™ phytochemicals, the results showed that 11 compounds had higher GOLD docking scores than EFV docked in HIV-1 RT (Table 2). These Kerra™ phytochemicals might be responsible for the anti-RT activity against HIV-1 RT.

### 2.3. Physicochemical and Pharmacological Profiling

The Kerra™ phytochemicals with higher GOLD docking scores than EFV were chosen for predicting their physiochemical and pharmacological profiles. Biochanin A, which had a GOLD score close to that of EFV, was also selected. These 12 phytocompounds were submitted to the SwissADME website to predict the physicochemical and ADME properties of the selected Kerra™ phytochemicals (Table 3 and Table 4). The phytochemicals had molecular weights between 254.41 and 504.48 daltons. The numbers of rotatable bonds, H-bond acceptors, and H-bond donors were between 2 and 15, 2 and 11, and 1 and 6 bonds, respectively. The rotatable bonds, total H-bond count (sum of donors and acceptors), and polar surface area can be used to predict the conformational flexibility and bioavailability of compounds [30]. Compounds with good bioavailability will have a polar surface area (PSA) of less than 140 Å^2^, a sum of H-bond acceptors and donors of less than 12, and number of rotatable bonds of less than 10 [30]. In the current study, 10 of the selected Kerra™ compounds fell within these criteria.

The lipophilicity, solubility, gastrointestinal (GI) absorption, blood–brain barrier (BBB) permeant, P-glycoprotein (P-gp) substrate, and cytochrome 450 inhibitor were pharmacological indices used to predict the absorption, distribution, metabolism, and excretion (ADME) profiles of the Kerra™ phytochemicals. The lipophilicities of the selected Kerra™ phytochemicals were between 0.28 and 5.32. The topological polar surface areas (TPSAs) were between 37.30 and 187.12 Å^2^ (Table 4). The ideal values of compounds should be 1–3 for lipophilicity and 20–130 Å^2^ for TPSA [31,32]. Five and eleven compounds fell within the ideal range of lipophilicity and TPSA, respectively. Six and six phytochemicals were predicted to be soluble and moderately soluble in water. Two, five, and six compounds had low GI absorption, were not BBB permeant, and did not have a Pgp substrate, respectively (Table 4). Nine compounds were cytochrome 450 inhibitors with different isoforms (Table 4).

In addition, the swissADME predicts the drug-likeness following Lipinski’s rule of five. The drug-like compounds should not have more than 5 H-bond donors, 10 H-bond acceptors, a molecular mass of 500 daltons, and a log P (octanol–water partition coefficient) of 5 [33]. The drug-likeness prediction of the selected Kerra™ phytochemicals is presented in Table 5, showing that 10 compounds exhibited drug-like properties. Consequently, these 10 compounds were searched for in chemical vendor databases for a subsequent in vitro inhibitory study.

### 2.4. Inhibition of Selected Kerra™ Phytochemicals against HIV-1 RT

**Figure 2:**
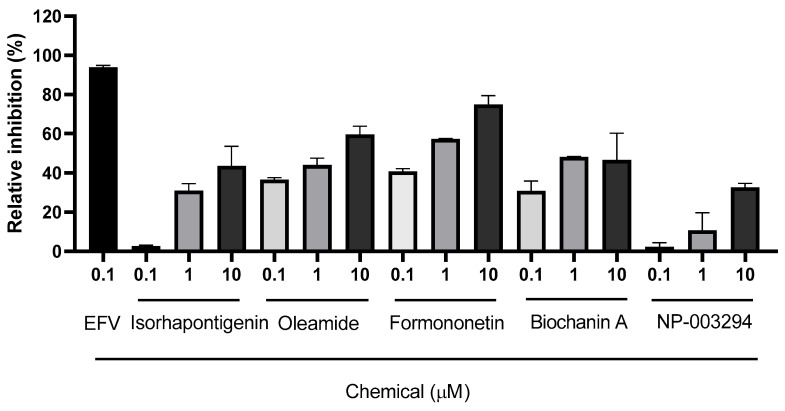
Relative inhibition percentages of efavirenz (EFV) and the 5 selected Kerra™ phytochemicals against HIV-1 RT.

**Figure 3:**
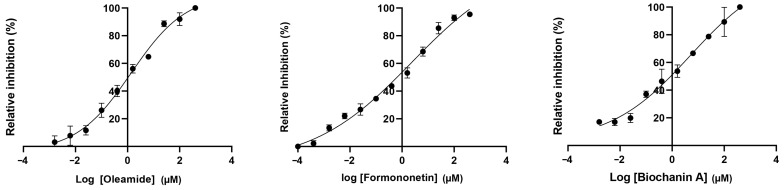
Non−linear regression dose–response curve of oleamide, formononetin, and biochanin A against HIV-1 RT.

Out of the 10 selected compounds, only 5 were obtained from the chemical vendors: isorhapontigenin, oleamide, formononetin, biochanin A, and NP-003294. Consequently, these five compounds were further tested for their inhibitory activity against HIV-1 RT. The result showed that these compounds had different relative inhibition values against HIV-1 RT (Figure 2). Isorhapontigenin and NP-003294, at concentrations of 10 µM, exhibited less than 60% relative inhibition. Therefore, these compounds were not selected for investigating their IC_50_ value against HIV-1 RT. The results revealed that oleamide had the lowest IC_50_ value, followed by formononetin, while biochanin A exhibited the highest IC_50_ value against HIV-1 RT (Figure 3 and Table 6).

**Figure 4:**
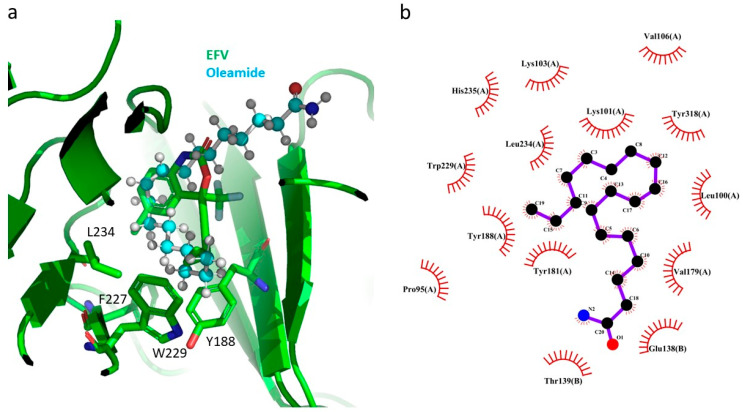
GOLD docking result for oleamide in HIV-1 RT. (**a**) The superimposition of the docking pose of oleamide (cyan/red/blue ball-and-stick representation) and EFV (green/red/blue stick representation) in NNBP of HIV-1 RT (green ribbon representation); (**b**) interaction analysis using the LigPlot program between oleamide (red/blue/black ball representation) and HIV-1 RT in NNBP.

Oleamide or cis-9-octadecenoamide has been found in natural resources [34,35]. Oleamide extracted from Zizyphus jujuba had an activation effect on choline acetyltransferase and neurotoxic inhibitory effects [35]. The current study is the first time that oleamide has been revealed to also possess antiviral activity. The docking pose analysis of oleamide in HIV-1 RT showed that the structural orientation of oleamide was similar to that of EFV (Figure 4a). The alkyl amide group of oleamide pointed toward the solvent-exposed surface of HIV-1 RT (Figure 4a). The alkyl group of oleamide was positioned in the hydrophobic tunnel of HIV-1 RT, which was surrounded by tunnel residues Y188, F227, W229, and L234 (Figure 4b). The intensive interaction between the hydrophobic tunnel and inhibitor has been shown to be crucial for the inhibition of HIV-1 RT [36]. The hydrogen bond presented at K101 residues has been shown to be conserved between RT and the inhibitors [36,37]. Although oleamide did not form this hydrogen bond, it had a hydrophobic interaction with C*γ* of K101 instead, which might compensate for the loss of this hydrogen bond [37]. These hydrophobic interactions might be an explanation of the inhibitory effect of oleamide against HIV-1 RT (Table 6). Additionally, the extensive interactions might explain why oleamide exhibited superior inhibitory activity compared to formononetin and biochanin A (Table 6).

**Figure 5:**
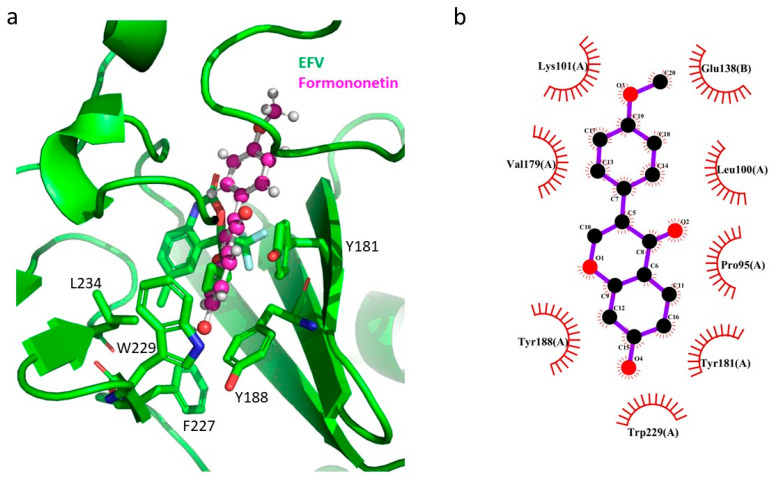
GOLD docking result for formononetin in HIV-1 RT (green ribbon representation). (**a**) The superimposition of the docking pose of formononetin (pink/red ball-and-stick representation) and EFV (green/red/blue stick representation) in NNBP of HIV-1 RT; (**b**) interaction analysis using the LigPlot program between formononetin (black/red ball representation) and HIV-1 RT in NNBP.

Formononetin is an O-methylated isoflavone from groups of phytoestrogens. It possesses various biological activities, such as antioxidant, anticancer, anti-inflammation, and antivirus [38,39]. Regarding its antivirus activity, it has been found that formononetin could reduce EV71 RNA and protein synthesis and could prevent EV71-induced cytopathic effects. It suppressed Cox-2/PGE2 expression, which were induced by EV71 infection [39]. In the current study, formononetin could inhibit HIV-1 RT. The docking result showed that formononetin had π–π stacking interactions with HIV-1 RT Y181 and W229 in the hydrophobic tunnel. It also had a π–anion interaction with E138 of the p51 subunit (Figure 5a). Formononatin also exhibited hydrophobic interactions with P95, L101, K101, V179, Y181, Y188, and W229 of p66 and E138 of p51 (Figure 5b). The hydrophobic interactions with several residues in both p66 and p51 might explain the inhibitory effect of formononetin against HIV-1 RT (Table 6).

**Figure 6:**
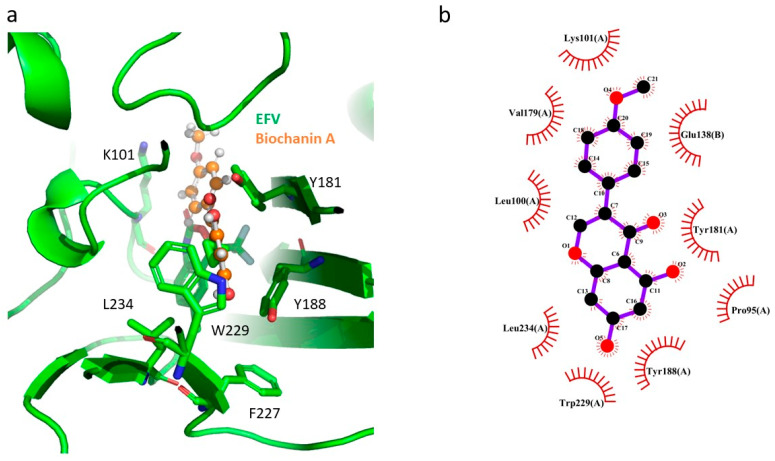
GOLD docking result for biochanin A in HIV-1 RT (green ribbon representation). (**a**) The superimposition of the docking pose of biochanin A (orange/red ball-and-stick representation) and EFV (green/red/blue stick representation) in NNBP of HIV-1 RT; (**b**) interaction analysis using the LigPlot program between biochanin A (black/red ball representation) and HIV-1 RT.

Biochanin A, an isoflavone found in soy, peanuts, and red clover, exhibits various biological activities, including anti-inflammatory, neuroprotective, antioxidant, antimicrobial, hepatoprotective, anticancer, and antiviral effects. [40,41]. It has been shown to inhibit the replication of H5N1 strains A/Thailand/Kan-1/04 and A/Vietnam/1203/04 [41]. In the current study, biochanin A demonstrated anti-HIV-1 RT activity. The docking study revealed that biochanin A formed a π–π-stacking interaction with residues Y181 and W229 (Figure 6a) and had hydrophobic interactions with several residues in NNBP residues, such as P95, L101, K101, V179, Y181, Y188, W229, and L234 of p66, as well as E138 of p51 (Figure 6b). The extensive interactions between NNBP residues and biochanin A might account for its inhibitory activity against HIV-1 RT (Table 6).

### 2.5. Inhibition of Pseudo-HIV Viral Infection

**Figure 7:**
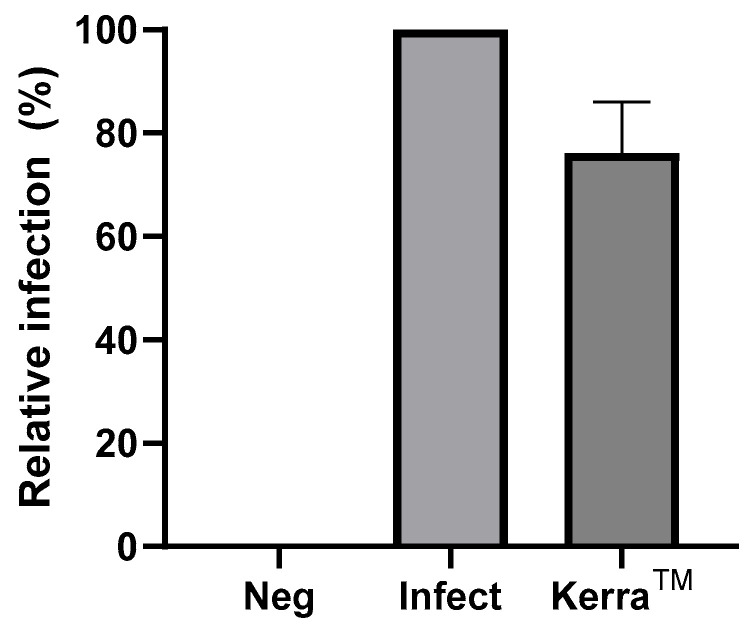
Percentage of the relative infection of pseudo-HIV virus into the HEK293 cells. Data are represented as the mean ± SD (*n* = 3). Neg, uninfected cells; Infect, infected cells with pseudo-HIV virus; Kerra™, the pseudo-HIV virus was incubated with 10 µg/mL Kerra™ extract before performing infection.

The inhibition of pseudo-HIV infection by NVP and EFV was previously achieved in our laboratory, and the results showed that NVP and EFV at a concentration of 20 µM had relatively low inhibitory activities, with relative infections of 97.64% and 81.55%, respectively [28]. The inhibition of pseudo-HIV infection using the Kerra™ extract was achieved in the present study, and is shown in Figure 7. The uninfected and pseudo-HIV-virus-infected cells were used as a negative (Neg) and positive infection control (Infect), respectively. Kerra™ at a concentration of 10 µg/mL could decrease pseudo-HIV virus infection into the HEK293 cells, with a relative infection of 76.12%, which inhibited viral infection more effectively than in our previous study on NVP and EFV [28]. Moreover, these results indicated that Kerra™ phytochemicals could pass through the cell membrane of HEK293 cell and inhibit HIV-1 replication in the HEK293 cell.

Based on these results, the phytochemicals in Kerra™, e.g., oleamide, formononetin, and biochanin A, could bind to the NNBP of HIV-1 RT, leading to the inhibitory activity of the Kerra™ extract against HIV-1. However, compared to EFV (Table 6), the inhibitory activity of the Kerra™ extract was lower. Nonetheless, Kerra™ contains various bioactive phytochemicals that may have synergistic effects in inhibiting HIV-1. It has been shown that the methanolic crude extract of *M. cochinchinensis* could inhibit HIV-1 protease [26]. Pterostilbene from *P. santalinus* L.f. was found to prevent HIV-1 infection in resting CD4 T cells at the reverse transcription step [25]. It also showed anti-HIV-integrase activity [42]. Isorhapontigenin was discovered to inhibit productive infection of HIV-1 at a concentration of 30 μM [42]. The use of NNRTIs and protease inhibitors in an ART regimen can cause various adverse effects, such as an allergic reaction, rash, insomnia, nausea, abdominal pain, vomiting, and hepatotoxicity [6]. It has been shown that formononetin and biochanin A could reduce hepatotoxicity induced by the HIV-1 protease inhibitor ritonavir [43]. In addition, Kerra™ has been shown to have low toxicity, with a CC_50_ value that is higher than 500 µg/mL, against Vero cells. [13]. Thus, Kerra™ can be consumed at a higher dose than other drugs. HIV infection can cause chronic inflammation [44]. After HIV infection, the virus causes a reduction in CD4, followed by the disruption of tight junctions in the intestinal epithelium and an imbalance in the intestinal microbiota composition. This leads to the release of bacterial products into the circulation, inducing chronic immune activation and inflammation. Chronic activation produces inflammatory biomarkers such as interleukin (IL)-6, IL-1β, and tumor necrosis factor (TNF)-α [44]. Seetaha et al. (2022) revealed that Kerra™ exhibited anti-inflammatory activity [12]. Its phytochemicals, such as 2-methoxy-9H-xanthen-9-one, betaine, isorhapontigenin, formononetin, and biochanin A, exhibited anti-inflammatory activities. Treatment with 5 and 15 µM isorhapontigenin in LPS-induced inflammation in RAW264.7 murine macrophage cells reduced the expressions of IL-6, IL-1β, and TNF-α, indicating the anti-inflammatory effects of isorhapontigenin [45]. Biochanin A shows anti-inflammatory effects via inhibition of IL-1β and TNF-α [40]. From these results, the phytochemicals in Kerra™ extract not only inhibit HIV-1 RT and pseudo-HIV-1 infection but also reduce the inflammation caused by HIV-1 infection. Further research and clinical trials are necessary to fully elucidate the therapeutic potential of Kerra™ in the context of HIV treatment.

## 3. Materials and Methods

### 3.1. Preparation of Kerra™ Extract

The Thai herbal medicine Kerra™ was kindly provided by VEJCHKORN MEDICINE REG.ORD.PART, Bangkok, Thailand. The Kerra™ extract was prepared by the maceration extraction method. One hundred grams of Kerra™ powder were incubated with 500 mL of 99.5% (*v*/*v*) ethanol (RCI Labscan, Bangkok, Thailand) (Kerra™:Ethanol, 1:5) at 25 °C, 150 rpm overnight. Then, the extract was filtered through Whatman no.1 filter paper (GE Healthcare, Buckinghamshire, UK) and was concentrated by a rotary evaporator (Evaporator model R 300, Buchi, Basel, Switzerland) at 45 °C. The obtained powder from the extraction process was dissolved in 100% DMSO (Loba Chemicals, Mumbai, India) to a final concentration of 100 mg/mL before use.

### 3.2. Protein Expression and Purification

Protein expression and purification of HIV-1 RT were performed according to [46]. Briefly, the protein was expressed from *Escherichia coli* BL21(DE3)-RIL harboring an RT p51 or RT p66 subunit. After that, protein expression was induced by using 0.5 mM IPTG at 16 °C for 16–18 h. The p51- and p66-expressing cells were collected by centrifugation at 5000 rpm, 4 °C for 15 min and were mixed in lysis buffer (50 mM Tris-HCl, pH 8.0, 300 mM NaCl, 10 mM imidazole, 5% glycerol, 0.5%Triton X-100). The cell mixture was centrifuged at 12,000 rpm, 4 °C for 20 min to collect the supernatant before subjecting it to Ni affinity chromatography, Resource S cation exchange, and Superdex 200 size exclusion chromatography, respectively, for RT purification. The purified HIV-1 RT was kept at −80 °C for further experiments.

### 3.3. Relative Inhibition Study and Determination of IC_50_

The relative inhibition study of HIV-1 RT was performed by using the fluorometric method [47]. An EnzChek^®^ reverse transcriptase assay kit (Molecular Probes, Eugene, OR, USA) was used. Then, 2 microliters of 12.5 mg/mL Kerra™ extract was mixed with 13 µL RT reaction buffer (50 mM TE pH 7.6, 2 mM DTT, 20% glycerol) in 384-well plates, and 5 µL of 50 nM purified HIV-1 RT was added. The reaction was started by adding 5 µL of the 1:400 primer/template substrate. The reaction was incubated at 37 °C for 30 min. The reaction was stopped by adding 5 µL of 0.2 M EDTA. Forty microliters of 1:700 Picogreen was added in the stopped reaction. Then, the reaction was incubated in the dark for 5 min, and the fluorescence was measured at excitation and emission wavelengths of 485 and 535 nm, respectively, using a microplate reader (Infinite F200 PRO, Tecan, Männedorf, Switzerland). Two microliters of 100% DMSO and 0.2 M EDTA were used instead of Kerra™ extract to serve as the positive control and background, respectively. The relative inhibition activities of the FDA-approved non-nucleoside reverse transcriptase inhibitors (NNRTIs), which were nevirapine (NVP), efavirenz (EFV), and rilpivirine (RPV), were also determined. Then, 2 microliters of 12.5 µM of each NNRTI was mixed with 13 µL RT reaction buffer in 384-well plates. After that, 5 microliters of 50 nM purified HIV-1 RT was added. The reaction was performed, and the fluorescence intensity was measured as described above. Three independent experiments were conducted for the Kerra™ extract and each NNRTI. The percentage of relative inhibition (%RI) was calculated from the following equation:

$$\%RI=\frac{\left(RTpositive-RTbackground\right)-(RTsample-RTbackground)}{\left(RTpositive-RTbackground\right)}\times 100$$

where RTpositive, RTbackground, and RTsample represented the fluorescence intensities of reactions containing 100% DMSO, 0.2 M EDTA, and Kerra™ extract, respectively. The data were analyzed by one-way ANOVA in GraphPad Prism 8 software (GraphPad Software Inc., La Jolla, CA, USA). The significant difference between groups was estimated by *p* < 0.05. Post hoc test was estimated by Bonferroni correction.

For IC_50_ determination, 9–10 concentrations of Kerra™ extract were prepared, ranging from 1.95 to 1000 µg/mL. The inhibition reaction of each concentration was performed as described above. The obtained relative inhibition values were fitted with the non-linear regression dose–response curve, which was generated using the GraphPad Prism 8 software.

### 3.4. Inhibition of Pseudo-HIV Viral Infection

To evaluate whether the Kerra™ extract could inhibit viral infection, the inhibition of pseudo-HIV viral infection by the Kerra™ extract was measured following the method of [28]. The lentivirus system was used to generate the pseudo-HIV virus in HEK293 cells. The pseudo-HIV virus was propagated from HEK293T, which was previously transfected with pfNL43 and pMD2.G plasmids [28]. Once the pseudo-HIV virus was obtained, it was incubated with 10 µg/mL Kerra™ extract for 1 h before incubated with HEK293 cells, which were previously seeded in 6-well plates at a density of 5 × 10^5^ cells/well overnight. After 1 h of incubation, the infected cells were amended with DMEM complete medium and incubated at 37 °C and 5% CO_2_ for 72 h. After that, the infected cells were harvested. The viral DNA was extracted, and the percentage of pseudo-HIV virus infection was determined by qPCR with the HIV LTR-specific primers [48].

### 3.5. In Silico Approach

The three-dimensional structure of HIV-1 RT was obtained from PDB ID 1FK9, which is the crystal structure of HIV-1 RT in a complex with the drug efavirenz (EFV) at the non-nucleoside binding pocket (NNBP) of the enzyme.

The phytochemicals of Kerra™ extract had been identified [12]. The chemical structures of the top 31 abundant phytochemicals found in the Kerra™ extract were searched for against the PubChem (https://pubchem.ncbi.nlm.nih.gov/, accessed on 27 December 2023) and AnalytiCon Discovery (https://ac-discovery.com/, accessed in 27 December 2023) databases, and 21 structures were obtained from these databases.

The interactions between the RT enzymes and the 21 phytochemicals found in the Kerra™ extract were studied by using the GOLD docking program [49]. The docking parameters were validated by re-docking the ligand EFV back into HIV-1 RT (1FK9). Once the docked ligand was in the same position as the ligand in the crystal structure, the same parameters were used in the docking of 18 Kerra™ phytochemicals into the NNBP of HIV-1 RT, as well as for the docking of the FDA-approved drugs, NVP, EFV, and RPV, into the NNBP of all RTs.

The prediction of physiochemical and pharmacological (adsorption, distribution, metabolism, and excretion; ADME) properties was performed by using SwissADME [32]. The canonical Simplified Molecular Input Line Entry Specification (SMILES) formats of the selected Kerra™ compounds were submitted onto the web (http://www.swissadme.ch/index.php, accessed on 3 January 2024). The drug-likeness (Lipinski’s rule of 5) was also predicted using SwissADME.

## 4. Conclusions

This study has demonstrated that the Thai herbal extract, Kerra™, exhibits significant anti-HIV-1 activity. Through in vitro assays, Kerra™ was shown to inhibit HIV-1 reverse transcriptase (RT) effectively, with a notable percentage of relative inhibition and IC_50_ value. Moreover, the reduction in pseudo-HIV infection in HEK293 cells highlights the potential of Kerra™ as an anti-HIV-1 agent.

Furthermore, the docking studies provided insights into the molecular interactions between Kerra™ phytochemicals and HIV-1 RT, which are crucial for understanding the mechanism behind the observed inhibitory effects. Molecular docking revealed that oleamide, formononetin, and biochanin A might account for the inhibitory activity of the Kerra™ extract against HIV-1 RT. The physicochemical and pharmacological property predictions further support the drug-likeness of Kerra™ components, aligning with Lipinski’s rule of five. The docking studies provided insights into the molecular interactions between the Kerra™ phytochemicals and HIV-1 RT, suggesting a mechanism for their inhibitory effects. Further research and clinical trials are warranted to fully elucidate the therapeutic potential and safety of Kerra™ in the context of HIV treatment.

## Figures and Tables

**Table 1:** The IC_50_ values of the FDA-approved drug efavirenz, (EFV) and Kerra™ extract against HIV-1 RT.

Chemical	IC_50_ (µg/mL)
EFV ^§^	0.005 ± 0.001
Kerra™ ^¥^	38.00 ± 4.62

^§^ The IC_50_ values of EFV against HIV-1 RT were calculated from the reference [28] by converting the IC_50_ unit from nanomolar (nM) to microgram/milliliter (µg/mL). ^¥^ The IC_50_ values of Kerra™ extract against HIV-1 RT were from the current study. Data are represented as the mean ± SD (*n* = 3).

**Table 2:** GOLD docking scores of 3 drugs, NVP, EFV, and RPV, and 21 most abundant phytochemicals found in Kerra™ extract with HIV-1 RT.

Chemical	GOLD Docking Score
NVP	51.5294
EFV	57.9499
RPV	70.7706
2-Methoxy-9*H*-xanthen-9-one	52.5800
Isorhapontigenin	59.8827
Betaine	43.3009
14-Deoxy-11,12-didehydroandrographolide	34.6598
Anethole	43.1458
5,8,11,14-Eicosatetraynoic acid	74.9822
NP-003294	65.9677
*N*1-(3-Chlorophenyl)-2-[2-(trifluoromethyl)-4-quinolyl]hydrazine-1-carboxamide	59.5124
Choline	42.6724
(1*S*,4*S*,5*R*,10*S*,13*S*,17*S*,19*S*,20*R*)-10-hydroxy-4,5,9,9,13,19,20-heptamethyl-24-oxahexacyclo[15.5.2.0^1,18^.0^4,17^.0^5,14^.0^8,13^]tetracos-15-en-23-one	24.9749
NP-006862	63.9812
DL-Stachydrine	44.4101
Apigenin 7-*O*-glucuronide	64.6422
Palmitoleic acid	61.7137
11-(4-Chloroanilino)-2,3-dihydro-1*H*-cyclopenta[4,5]pyrido[1,2-a] benzimidazole-4-carbonitrile	58.4916
NP-009051	55.1730
Oleamide	60.0396
Formononetin	59.1999
(1*S*,4a*S*,7a*S*)-7-[[(*E*)-3-Phenylprop-2-enoyl]oxymethyl]-1-[(2*S*,3*R*,4*S*,5*S*,6*R*)-3,4,5-trihydroxy-6-(hydroxymethyl)oxan-2-yl]oxy-1,4a,5,7a-tetrahydrocyclopenta[c]pyran-4-carboxylic acid	60.7748
3′,4′-Dimethoxyacetophenone	43.5958
Biochanin A	56.4515

NVP, nevirapine; EFV, efavirenz; RPV, rilpivirine.

**Table 3:** Physicochemical properties of the 12 selected Kerra™ phytochemicals.

Chemical	Molecular Weight (Dalton)	Number of Rotatable Bonds	Number of H-Bond Acceptors	Number of H-Bond Donors
Isorhapontigenin	258.27	3	4	3
5,8,11,14-Eicosatetraynoic acid	296.40	6	2	1
*N*1-(3-Chlorophenyl)-2-[2-(trifluoromethyl)-4-quinolyl]hydrazine-1-carboxamide	380.75	6	5	3
Apigenin 7-O-glucuronide	446.36	4	11	6
Palmitoleic acid	254.41	13	2	1
11-(4-Chloroanilino)-2,3-dihydro-1*H*-cyclopenta[4,5]pyrido[1,2-a] benzimidazole-4-carbonitrile	358.82	2	2	1
Oleamide	281.48	15	1	1
Formononetin	268.26	2	4	1
(1*S*,4a*S*,7a*S*)-7-[[(*E*)-3-Phenylprop-2-enoyl]oxymethyl]-1-[(2*S*,3*R*,4*S*,5*S*,6*R*)-3,4,5-trihydroxy-6-(hydroxymethyl)oxan-2-yl]oxy-1,4a,5,7a-tetrahydrocyclopenta[c]pyran-4-carboxylic acid	504.48	9	11	5
Biochanin A	284.26	2	5	2
NP-003294	344.32	4	7	2
NP-006862	336.47	6	4	3

**Table 4:** In silico adsorption, distribution, metabolism, and excretion property analysis of the 14 selected Kerra™ phytochemicals.

Chemical	TPSA *	Lipophilicity (LogP_O/W_)	Water Solubility	GI Absorption ^¥^	BBB Permeant ^§^	Pgp Substrate ^¶^	CYP Inhibitor ^£^
Isorhapontigenin	69.92	2.63	Soluble	High	Yes	No	CYP1A2, CYP2C9, CYP3A4
5,8,11,14-Eicosatetraynoic acid	37.30	4.78	Moderately soluble	High	Yes	No	CYP1A2, CYP2C19, CYP2C9
*N*1-(3-Chlorophenyl)-2-[2-(trifluoromethyl)-4-quinolyl]hydrazine-1-carboxamide	66.05	3.94	Moderately soluble	High	No	No	CYP1A2, CYP2C19, CYP3A4
Apigenin 7-*O*-glucuronide	187.12	0.28	Soluble	Low	No	Yes	No
Palmitoleic acid	37.30	4.92	Moderately soluble	High	Yes	No	CYP1A2, CYP2C9
11-(4-Chloroanilino)-2,3-dihydro-1*H*-cyclopenta[4,5]pyrido[1,2-a] benzimidazole-4-carbonitrile	53.12	4.49	Moderately soluble	High	Yes	Yes	CYP1A2, CYP2C19, CYP2C9, CYP2D6, CYP3A4
Oleamide	43.09	5.32	Moderately soluble	High	Yes	No	CYP1A2, CYP2C9
Formononetin	59.67	2.66	Soluble	High	Yes	No	CYP1A2, CYP2D6, CYP3A4
(1*S*,4a*S*,7a*S*)-7-[[(*E*)-3-Phenylprop-2-enoyl]oxymethyl]-1-[(2*S*,3*R*,4*S*,5*S*,6*R*)-3,4,5-trihydroxy-6-(hydroxymethyl)oxan-2-yl]oxy-1,4a,5,7a-tetrahydrocyclopenta[c]pyran-4-carboxylic acid	172.21	0.33	Soluble	Low	No	Yes	No
Biochanin A	79.90	1.50	Soluble	High	No	Yes	CYP2D6, CYP3A4
NP-003294	98.36	2.54	Moderately soluble	High	No	No	CYP1A2, CYP2C9, CYP2D6, CYP3A4
NP-006862	77.76	3.26	Soluble	High	Yes	Yes	No

* Topological polar surface area, ^¥^ gastrointestinal absorption, ^§^ blood–brain barrier, ^¶^ P-glycoprotein substrate, ^£^ Cytochrome P450.

**Table 5:** Drug-likeness prediction of 14 selected compounds of Kerra™, determined by using swissADME.

Chemical	Drug-likeness
Isorhapontigenin	Yes
5,8,11,14-Eicosatetraynoic acid	Yes
*N*1-(3-Chlorophenyl)-2-[2-(trifluoromethyl)-4-quinolyl]hydrazine-1-carboxamide	Yes
Apigenin 7-*O*-glucuronide	No
Palmitoleic acid	Yes
11-(4-Chloroanilino)-2,3-dihydro-1*H*-cyclopenta[4,5]pyrido[1,2-a] benzimidazole-4-carbonitrile	Yes
Oleamide	Yes
Formononetin	Yes
(1*S*,4a*S*,7a*S*)-7-[[(*E*)-3-Phenylprop-2-enoyl]oxymethyl]-1-[(2*S*,3*R*,4*S*,5*S*,6*R*)-3,4,5-trihydroxy-6-(hydroxymethyl)oxan-2-yl]oxy-1,4a,5,7a-tetrahydrocyclopenta[c]pyran-4-carboxylic acid	No
Biochanin A	Yes
NP-003294	Yes
NP-006862	Yes

**Table 6:** The IC_50_ values of oleamide, formononetin, and biochanin A against HIV-1 RT.

Chemical	IC_50_ (µM)
EFV ^§^	0.016 ± 0.004
Oleamide	1.37 ± 0.30
Formononetin	2.65 ± 0.80
Biochanin A	4.73 ± 1.04

^§^ The IC_50_ value of EFV against HIV-1 RT is from our previous study [28].

## Data Availability

Data is contained within the article.
